# Highly Sensitive Plasmon Refractive Index Sensor Based on MIM Waveguide

**DOI:** 10.3390/mi15080987

**Published:** 2024-07-30

**Authors:** Wen Jiang, Shubin Yan, Xiaoran Yan, Aiwei Xu, Guang Liu, Chong Wang, Lei Li, Xiangyang Mu, Guowang Gao

**Affiliations:** 1 School of Electrical Engineering, Xi’an Shiyou University, Xi’an 710065, China; 2 School of Electrical Engineering, Zhejiang University of Water Resources and Electric Power, Hangzhou 310018, China; 3 Zhejiang-Belarus Joint Laboratory of Intelligent Equipment and System for Water Conservancy and Hydropower Safety Monitoring, Hangzhou 310018, China

**Keywords:** Fano resonance, plasmon refractive index nanosensor, finite element method (FEM), sensitivity, figure of merit (FOM)

## Abstract

This paper introduces a novel plasmon refractive index nanosensor structure based on Fano resonance. The structure comprises a metal–insulator–metal (MIM) waveguide with an inverted rectangular cavity and a circle minus a small internal circle plus a rectangular cavity (CMSICPRC). This study employs the finite element method (FEM) to analyze the sensing characteristics of the structure. The results demonstrate that the geometrical parameters of specific structures exert a considerable influence on the sensing characteristics. Simulated experimental data show that the maximum sensitivity of this structure is 3240 nm/RIU, with a figure of merit (FOM) of 52.25. Additionally, the sensor can be used in biology, for example, to detect the concentration of hemoglobin in blood. The sensitivity of the sensor in this application, according to our calculations, can be 0.82 nm∙g/L.

## 1. Introduction

Optical refractive index sensors are classified into several categories, including fiber optic sensors [1], grating sensors [2], micro-ring resonator sensors [3], and surface plasmon resonance (SPR) sensors. Photonic devices exhibit superior immunity to electromagnetic interference [4], operating bandwidth [5], long-distance transmission, and low power consumption in comparison to electronic devices [6]. Nevertheless, the optical diffraction limit impedes their miniaturization and integration, hindering the progress of optical refractive index sensors. However, SPR sensors operate on optical principles because they are capable of generating highly localized electromagnetic fields. This ability allows SPPs to somewhat exceed the diffraction limit of conventional optics [7]. This is the reason why SPPs are receiving increasing levels of attention.

Surface plasmon polaritons (SPPs) are distinctive electromagnetic waves that exist at the interface between a metal and a medium, such as air or water [8]. The generation of SPPs is contingent upon the interaction between free electrons within the metal and photons of incident light [9]. This interaction results in the propagation of the electromagnetic wave along the interface, with the field strength concentrated near the interface and exhibiting exponential decay in a direction perpendicular to it [10]. This property creates a highly localized light field at the interface, which permits the manipulation of light beyond the limits of conventional optical diffraction. This allows for the control and steering of light on a subwavelength scale [11]. These distinctive characteristics of SPPs facilitate the development of innovative technological solutions in a range of applications, particularly in the field of chip-scale photonic devices. In these applications, the localized electric field enhancement effect of SPPs is of critical importance, significantly improving device performance. This includes an enhanced light–matter interaction strength, which renders the system highly sensitive to localized structures on metal surfaces and refractive index alterations within the medium [12]. The fabrication of SPPS-based optical devices, including all-optical switches [13], filters [14], optical modulators [15], refractive index sensors, and other devices, is a viable proposition. These advances are of significant importance for data storage, spectroscopic measurements, and biosensing and provide a robust foundation for innovations in the fields of micro- and nanophotonics, information technology, and life sciences.

A significant proportion of structures have the capacity to energize the surface plasmon polariton (SPP) effectively. Of these, MIM waveguide structures have attracted significant attention in nanophotonics, especially in surface plasmon excitation technology, due to their ability to tightly bind light at subwavelength scales. MIM waveguides consist of two layers of metal with an insulating layer situated between them. They are capable of supporting the propagation of SPPs and enabling highly integrated optical device designs. In MIM waveguides, local resonance modes (e.g., resonance of nanocavities) couple to the propagation modes in the waveguide. This coupling results in the formation of a Fano resonance, characterized by an asymmetric spectrum, with the local resonance modes and propagation modes interfering with one another [16].

The generation of Fano resonance not only enhances the local electromagnetic field in the SPPs system but also improves the modulation and control of light in this system, which has important applications in optical sensing, spectroscopy, and optoelectronics [17]. By the meticulous engineering of metal nanostructures, the characteristics of Fano resonance, including resonance frequency, linewidth, and degree of asymmetry, can be tuned to meet specific application requirements. The introduction of specific nanostructures into MIM waveguides facilitates the formation of Fano resonances, resulting in the emergence of a distinctive Fano line pattern in the transmission or reflection spectrum. This pattern is characterized by the presence of pronounced resonance peaks or troughs superimposed on a broadband background. The high sensitivity of the Fano resonance makes the MIM waveguide structure well-suited for optical sensing applications, especially where detecting very small physical, chemical, or biological changes is necessary. By utilizing the sharp nature of the Fano resonance at specific frequencies, highly sensitive detection of environmental changes is possible [18,19].

Wang et al. designed a metal–insulator–metal (MIM) waveguide with two silver baffles coupled to a half-ring cavity. This structure achieved a maximum sensitivity of 2200 nm/RIU and was used as a temperature sensor with a sensitivity of 0.9 nm/°C [20]. Chen et al. proposed a metal–insulator–metal (MIM) waveguide consisting of two truncated resonators and a ring resonator. This design reached a maximum sensitivity of 1650 nm/RIU and could be used as a refractive index sensor and a stopband filter [21]. Zhu et al. proposed a unilaterally coupled metal–insulator–metal (MIM) structure using surface plasma and a U-shaped cavity with a sensing sensitivity of up to 825 nm/RIU [22]. Rashid et al. introduced a structure based on three circularly coupled straight metal–insulator–metal (MIM) waveguides. This structure exhibited a maximum sensitivity of 3573.3 nm/RIU and a quality factor of 21.9 [23]. Li et al. presented a structure consisting of a connected ring resonator (CRR) coupled to a MIM bus waveguide structure, achieving a maximum sensitivity of 1085 nm/RIU [24]. Zhang et al. proposed a symmetrically intersecting rectangular semi-annular cavity and a long, straight waveguide structure with a refractive index sensitivity of 1222 nm/RIU and a FOM of 35.16 [25]. Chen et al. designed a structure that includes a metal–insulator–metal (MIM) plasma waveguide and semi-elliptical and rectangular ring resonator cavities. This structure achieved a maximum sensitivity of 1384 nm/RIU and a quality factor (FOM) of 28.4 [26]. In this study, we propose a novel structure consisting of a MIM waveguide with an inverted rectangular cavity, the circle minus a small internal circle plus a rectangular cavity (CMSICPRC). The sensitivity of the CMSICPRC structure is significantly improved through linear regression analysis to calculate the slope, achieving a maximum sensitivity of 3240 nm/RIU and a quality factor (FOM) of 52.25. The geometrical parameters of the structure can be adjusted for different scenarios. We used it to simulate the detection of the hemoglobin concentration in blood, and according to our calculations, the sensor’s sensitivity can reach 0.82 nm∙g/L, demonstrating excellent potential sensitivity in the field of biomedicine.

## 2. Materials and Methods

**Figure 1:**
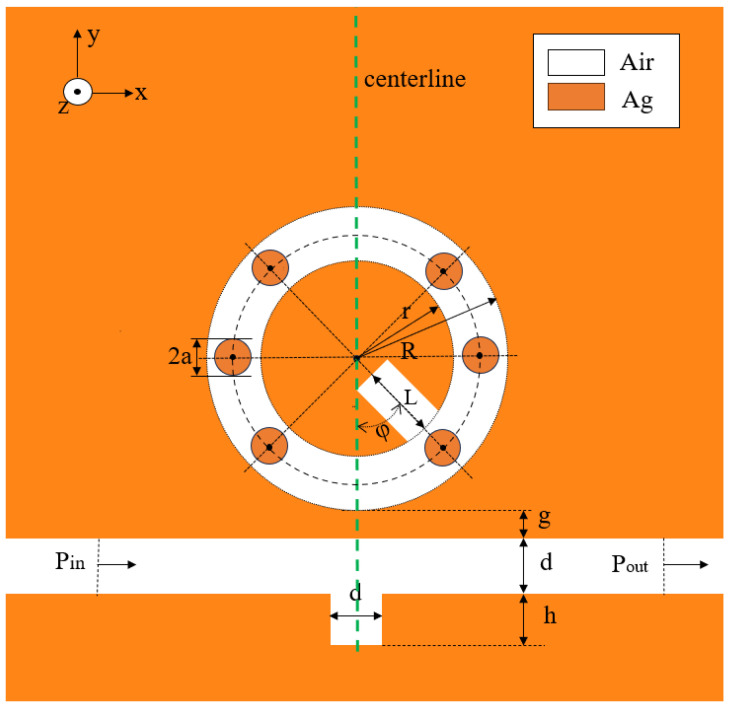
Schematic two-dimensional (2D) layout of the designed sensor fabric.

In this study, the finite element method is employed to initially examine the influence of a change in refractive index on the Fano resonance. Subsequently, the geometrical parameters of the designed structure were adjusted in order to observe their effect on the Fano resonance. The SPP surface depth represents only a minor portion of the overall results set. While simulating a three-dimensional model necessitates more sophisticated hardware and meticulous meshing, the magnetic field properties of two-dimensional and three-dimensional models are strikingly similar. To facilitate the calculations, we elected to simulate a two-dimensional (2D) model [27]. The variation in resonance frequency and intensity with respect to the refractive index was observed, and the subsequent analysis focused on the magnetic field distribution. The majority of designed toroidal cavity-based structures consist of toroidal cavities plus one or two circles or polygons in a parallel set. However, the results of this study demonstrate a notable enhancement in the sensitivity and quality factors through the utilization of a distinctive set based on a toroidal cavity structure with a small circle, as shown in Figure 1. The figure shows a symmetric structure along the centerline, consisting of a MIM waveguide with an inverted rectangular cavity and a circle minus a small internal circle plus a rectangular cavity (CMSICPRC). The center of the small circle is positioned at the horizontal and plus or minus 45-degree lines. The parameters are defined as follows: the radius of the outer circle of the CMSICPRC structure is R, the radius of the inner circle is r (R − r = 50 nm), the widths of the waveguide and the two rectangles are d (d = 50 nm), the length of the rectangle within the circle is L, the length of the inverted rectangular waveguide is h, the radii of the individual small circles are a, and g is the coupling distance between the MIM waveguide and the CMSICPRC structure.

The propagation of electromagnetic waves at the metal–dielectric interfaces propagate in two main modes: transverse electric (TE) and transverse magnetic (TM). The TE mode has an electric field perpendicular to the direction of propagation and is parallel to the interface, while the TM mode has a magnetic field perpendicular to the direction of propagation and is parallel to the interface. The orientation of the electric and magnetic fields of each mode with respect to the propagation direction is well characterized [28]. SPPs constitute a distinct category within the TM mode, emerging from the interaction between electromagnetic waves and metal-free electrons at the metal–dielectric interface. SPPs propagate along the metal–dielectric interface at a fixed angle to the direction normal to the interface, and their electric and magnetic fields satisfy the conditions of the TM mode. Since SPPs possess the TM property, they can localize the electromagnetic field at the metal–dielectric interface and produce an electromagnetic field enhancement on the subwavelength scale. This localized EM field fluctuation is crucial for many applications, such as photonic devices and sensors. In contrast, TE modes cannot form similar localized fluctuations at metal–dielectric interfaces and thus cannot characterize the phenomenon of surface plasmon excitations. Therefore, our analysis focuses on the TM mode, with a particular focus on the relationship between SPPs and dispersion [29]:

(1) $$\tanh\left(k\omega \right)=-\frac{2k\alpha_{c}}{k^{2}+p^{2}\alpha_{c}}$$

where *k* expresses the wave vector, and in vacuum, $k_{0}=2\pi /\lambda_{0}$; $\alpha_{c}^{2}=k_{0}^{2}\left(\varepsilon_{in}-\varepsilon_{m}\right)+k^{2}$ and $p=\varepsilon_{in}/\varepsilon_{m}$; Among them, $\varepsilon_{in}$ and $\varepsilon_{m}$ are, respectively, the permittivity of the insulator and metal.

The width of the dielectric layer in metal–insulator–metal (MIM) waveguides significantly affects waveguide performance. The width of the dielectric layer (d) is a key parameter because it directly affects the types of modes the waveguide can support and their limiting conditions. At a specific dielectric layer width, e.g., 50 nm, the propagation conditions for transverse magnetic (TM) modes can be optimized, and the presence of transverse electric (TE) modes may be reduced or eliminated [30].

In Figure 1, the green area denotes silver, while the white area signifies air. Silver (Ag) exhibits the lowest damping constant *Γ* among metals and excels at optical frequencies [31]. Due to its superior optical properties, nanostructured silver is ideal for next-generation plasma waveguides. It offers nanoscale beam confinement, effective optical waveguiding, imaging capabilities, and low propagation losses [32]. Considering the varying dielectric constant of metals with frequency, we calculated the relative permittivity of silver using the Drude–Debye model (the permittivity of air is 1) [33].

(2) $$\varepsilon \left(\omega \right)=\varepsilon_{\infty }+\frac{\varepsilon_{s}-\varepsilon_{\infty }}{1+i\omega \tau }+\frac{\sigma }{i\omega \varepsilon_{0}}$$

The relative permittivity of infinite frequencies $\varepsilon_{\infty }$ is 3. 8344.The static dielectric coefficient $\varepsilon_{s}$ is −9530.5, and the relaxation time (τ) is $7.4\times 10^{-15}$ seconds. In addition, the conductivity is $1.1486\times 10^{7}\;s/m$ and the angular frequency (ω) =$1.38\times 10^{16}\;rad/s$.

In this study, three key parameters are employed to assess the performance of the sensor: sensitivity (S), full width at half maximum (FWHM), and figure of merit (FOM) [34]. Sensitivity, a key property of sensors, of the MIM waveguide structure based on Fano resonance is determined by the shift in wavelength position. The formula for sensitivity is provided below:

(3) $$S=\frac{\Delta \lambda }{\Delta n}$$

Fano resonance is characterized by a sharp peak in the transmission spectrum; the sharper this peak, often the *FWHM* (full width at half maximum), is smaller. A smaller *FWHM* indicates better performance. Specific definitions are provided below [35]:

(4) $$FWHM=\lambda_{1}-\lambda_{2}$$

$\lambda_{1}and\;{\;\lambda }_{2}$ are the two wavelengths corresponding to the resonance curves at one-half transmittance of $T_{max}$ and $T_{min}$. $T_{min}$ and $T_{max}$ denote the minimum and maximum transmittance in the transmission spectrum.

The *FOM* is defined as follows [36]:

(5) FOM=S/FWHM.

Higher *FOM* values indicate higher sensitivity or a narrower half-height width.

## 3. Results

**Figure 2:**
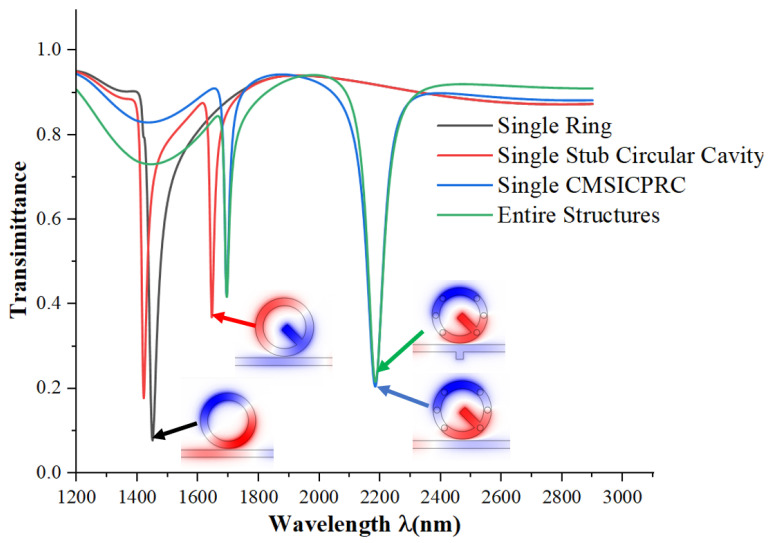
Transmission spectra of single ring, single stub circular cavity, single CMSICPRC, and all systems.

We used COMSOL Multiphysics 5.4a software to simulate the model and analyze the propagation characteristics using the FEM. Since Fano resonance can effectively respond to the sensor’s performance, we mainly analyzed its generation and characteristics. We set the parameterized scanning interval and size from 1200 nm to 2900 nm, with an interval size of 1 nm: the smaller the interval, the larger the calculation volume, and the more accurate the results. First, we analyzed the classical waveguide-coupled circular resonant cavity, which has a cavity width of 50 nm. From the transmission spectrogram in Figure 2, we observed a sharp valley, and the transmission spectrum of the complete structure is an asymmetric curve, indicating that the whole system excites Fano resonance. Next, we simulated the structure of a single stub circular cavity (SSCC) and found it has two troughs with different transmittances. The working wavelength range is on both sides of the circular resonant cavity. We then subtracted six small circles with symmetric radii of 20 nm around the centerline from the single short-stub circular cavity. This also resulted in two troughs but with larger working wavelength ranges than the previous structures. Finally, we added a short stub with a length of 50 nm to the waveguide and found that the trough position of the transmission spectra remained almost unchanged. The FWHM at 1750 nm broadened, and the FWHM at 2200 nm narrowed.

**Figure 3:**
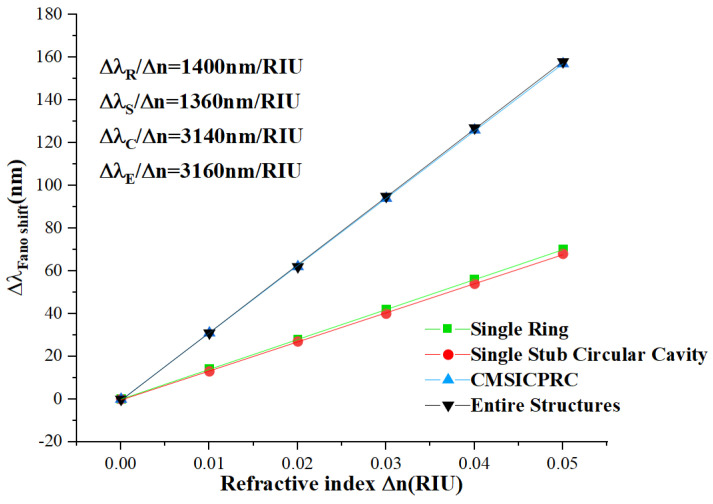
Sensitivity fit lines for different structures.

Subsequently, the experiments were conducted to determine the refractive index values, ranging from *n* = 1.00 to *n* = 1.05. An interval of 0.01 was chosen to demonstrate that a change in refractive index leads to a change in resonance wavelength. This interval was arbitrarily chosen for the evaluation of the sensor structure. We calculated the sensitivity performance parameters for each of the four structures. Since the last three structures have two different troughs, we calculated them separately. Through linear regression analysis to calculate the slope, as we can see from Figure 3, the sensitivity of the circular resonant cavity is 1400 nm/RIU. The sensitivities of the high transmittance (high trough) for the last three structures are 1660, 1720, and 1725 nm/RIU, respectively. The sensitivities of the low transmittance (low trough) are 1360, 3140, and 3160 nm/RIU, respectively. Due to the greatly improved sensitivities of our proposed structures, the transmittances at the high troughs are comparatively high, and the sensitivities are low. Accordingly, our attention was directed towards the transmission spectra of low transmittance.

Eventually, we determined the prototype of the structure. In addition to the refractive index affecting the Fano resonance, the specific parameters of the structure have an important effect. To gain a deeper understanding of the impact of specific parameters on the transmission spectra, we varied the geometrical parameters of the structure and observed the resulting Fano resonance transmission spectra.

**Figure 4:**
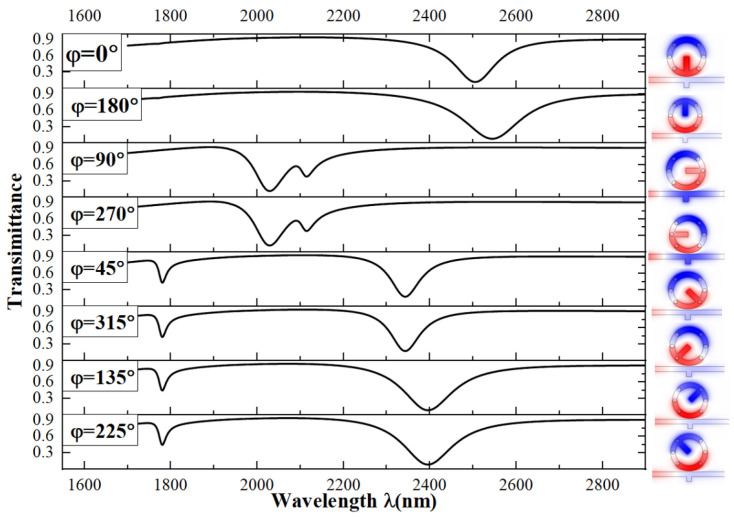
Transmission spectra of a single short rod in a circle at different angles.

First, we adjusted the angle φ of the single short bar inside the circle, varying φ from 0° to 315° in steps of 45°. The results are shown in Figure 4. We found that the transmission spectra are similar in pairs. When the angls are 0° and 180°, the transmission spectrograms are more similar. There is only one resonance valley from 1700 nm to 2900 nm, with the wavelength near 2500 nm. The calculated sensitivities are 2330 and 2367 for 0° and 180°, respectively. When the angles are 90° and 270°, the transmission spectra are very similar. There are two valleys, but they are close to each other, making the working wavelengths too close to show good sensor performance. Therefore, we do not consider the results for these two angles. We observed that the troughs at angles 45° and 315° are a bit sharper than those at 135° and 225°, indicating a better Fano resonance. We then plotted the transmission spectra for 45°, 135°, 225°, and 315° together in Figure 4 and found that they are almost the same with only minor differences. The sensitivities at 45° and 135° are calculated to be 3160 and 3360, respectively, while the FOMs are 50 and 32, respectively. Although the sensitivity at 135° is 0.063 higher than that at 45°, the FOM value is 0.56 lower. Therefore, we decided to study the structure with the short bar at 45°.

**Figure 5:**
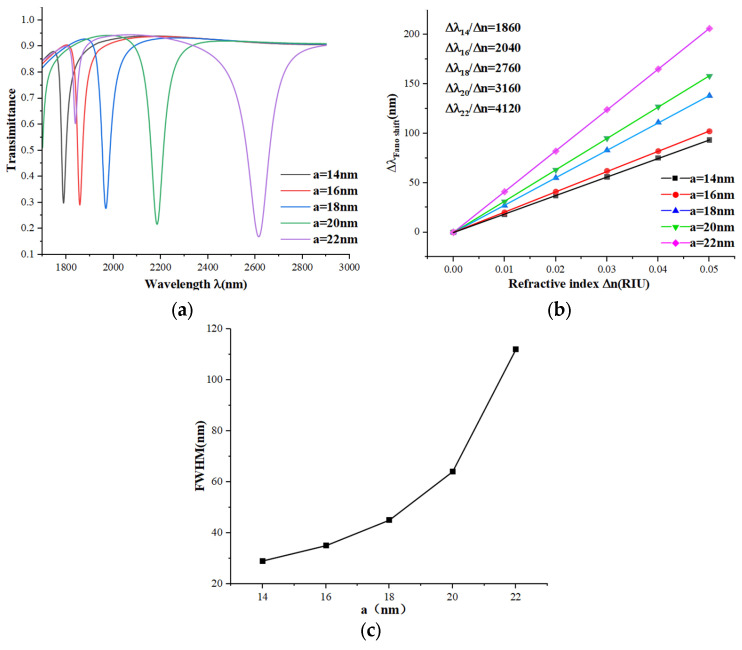
(**a**) Transmission spectra of different radii a; (**b**) sensitivity fitted lines of different radii a; (**c**) variation in FWHM values of different radii a.

Next, we modeled the effect of the radius of the circle inside the ring (a) on the performance of the sensor based on the simulation and computational results. We set the radii of a to 14, 16, 18, 20, and 22 nm, and the generated spectrograms are shown in Figure 5a. It is clear that the transmission spectrum undergoes a larger redshift and moves downward as a becomes larger, as seen in Figure 5b. As the radius increases, the sensor’s sensitivity also increases with larger step sizes. However, Figure 5c shows that the FWHM gradually expands, with the FWHM at 22 nm being almost double that at 20 nm. The FOM for 22 nm is calculated to be only 70% of that for 20 nm. To summarize, a circle radius of 20 nm provides the best overall performance.

**Figure 6:**
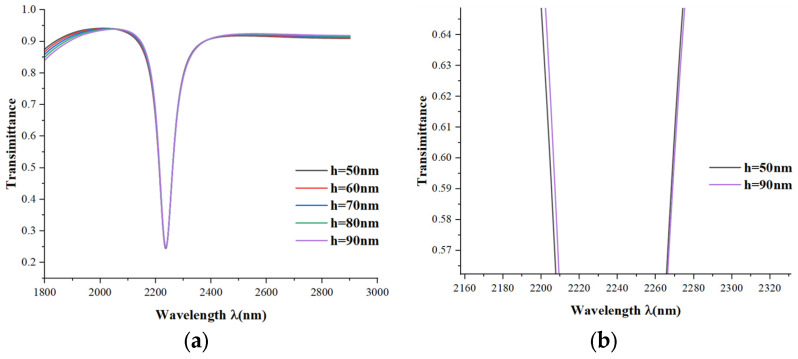
(**a**) Transmission spectra for different lengths h; (**b**) detail view at ($T_{max}$+ $T_{min}$)/2.

After that, we modeled the effect of length h of the inverse rectangle on the performance of the sensor based on the simulation and computational results. We increased the length h from 50 to 100 nm in steps of 10 nm, as shown in Figure 6. When the length changes slightly, the wavelengths of the transmission spectra and the Fano resonance remain almost unchanged; when we display the detailed images for h = 50 nm and h = 90 nm at ($T_{max}$+ $T_{min}$)/2, we calculated that the FWHM is 1 less for h = 90nm compared to h = 50 nm. Therefore, the FOM at h = 90 nm is slightly improved.

**Figure 7:**
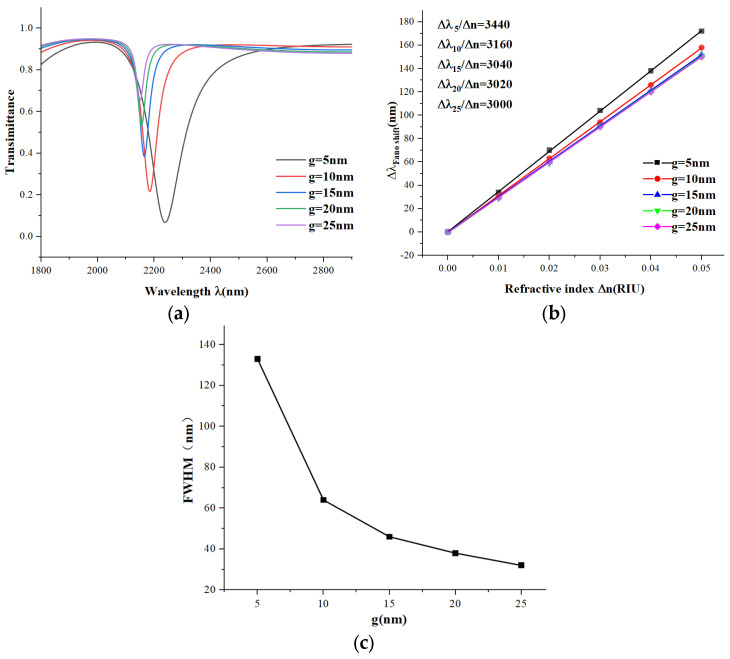
(**a**) Transmission spectra at different distances g; (**b**) sensitivity fit lines at different distances g; (**c**) variation in FWHM values at different distances g.

Next, we studied the effect of the coupling distance g between the waveguide and the entire structure. We increased the coupling distance g from 5 nm to 25 nm in steps of 5 nm. From Figure 7a, it is clear that as the coupling distance increases, the transmission spectra are blue-shifted, and the trough position shifts upward. The transmittance also increases with g, indicating that the coupling effect and the electric field strength are weakening. Figure 7b shows that as the coupling distance increases, the sensitivity gradually decreases, but the rate of reduction diminishes. Figure 7c shows that from 5 to 10 nm, the FWHM steeply reduces by more than half, and then the reduction rate decreases. Based on this analysis, we choose 10 nm as the coupling distance for a relatively good low transmittance performance.

**Figure 8:**
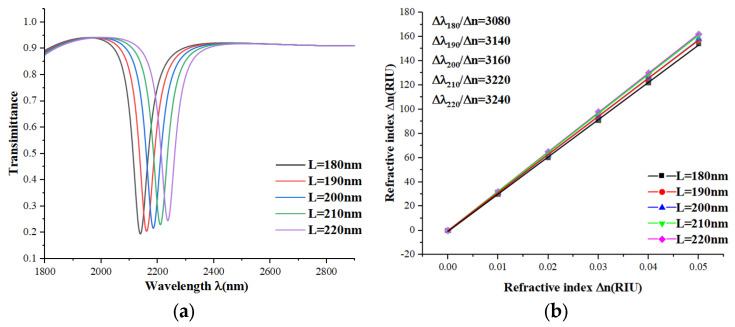
(**a**) Transmission spectra for different lengths of L; (**b**) sensitivity fit lines for different lengths of L.

Next, to study the effect of specific parameters more deeply, we adjusted the length L of the single short bar from 180 to 220 nm in steps of 10 nm without changing the other parameters. From the transmission spectra plots in Figure 8a and the sensitivity fitting curves in Figure 8b, we can see that the transmission spectra undergo a slight equidistant redshift and shift upward as the length increases, with the sensitivity becoming slightly larger, but the FWHM remains almost the same. From this, we conclude that the length of the single short bar is also a factor affecting the sensor parameters. Within the range of 180 to 220 nm, the longer the single short bar, the better the sensor performance.

**Figure 9:**
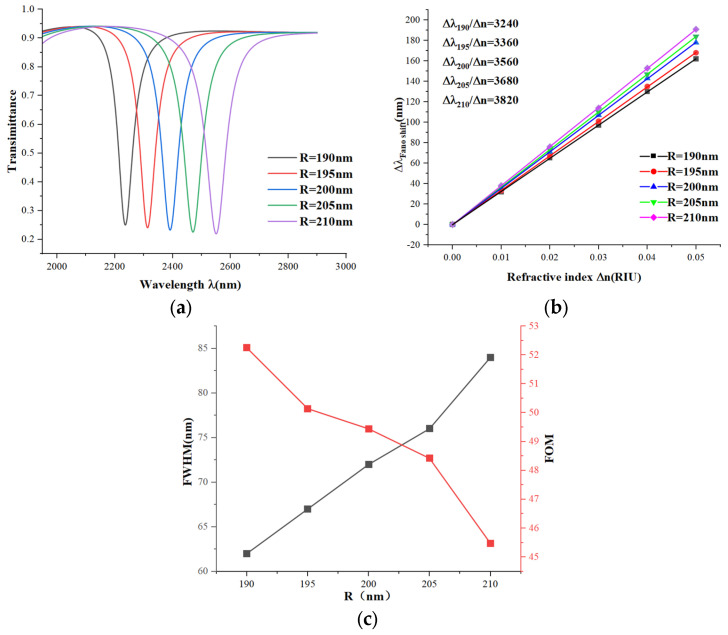
(**a**) Transmission spectra of different radii R; (**b**) sensitivity fitted lines of different radii R; (**c**) variation in FWHM and FOM values of different radii R.

Finally, we modeled the effect of the large circular radius R on the performance of the sensor based on the simulation and computational results. (always keeping R − r = 50 nm). We start with a large circular radius R = 190 nm, which is increased in steps of 5 nm up to 210 nm. We can see from Figure 9a that as the radius R increases, the position of the wave trough is red-shifted, and the sharpness decreases, and from Figure 9b, the sensitivity increases as the radius R increases. But, as can be seen from Figure 9c, as the radius R increases, the FWHM increases and the FOM decreases. Based on this analysis, the sensor has the best overall performance when the radius R is 190 nm.

## 4. Biological Sensing Applications

Due to the sensing properties of this structure, the sensor utilizes the surface plasmon resonance generated by the interaction of light with electron waves on the metal surface to detect changes in the refractive index. Its high sensitivity to small changes in the refractive index allows for molecular detection at the nanomolar level and real-time monitoring without the need for labeling or staining the sample. The sensor model can be used for biosensing, with refractive indices of biological samples ranging from 1.33 to 1.40. The input of the biosensor based on this design will be connected to a nanofiber, providing a pathway for the incident light, and the output will be connected to a JY confocal Raman microscope to detect the output signal. Therefore, we can apply it to human health testing, such as detecting the hemoglobin concentration in blood.

Red blood cells are one of the most common cell types in the human body, and their main function is to carry oxygen and carbon dioxide. The key to the ability of red blood cells to perform this function is that they contain high levels of hemoglobin. Hemoglobin is an important indicator in blood testing. It provides clinicians with crucial information about a patient’s blood and overall health status to guide further diagnosis and treatment. This includes the diagnosis and classification of anemia, assessment of blood disorders, monitoring of bone marrow function, diagnosis and monitoring of hemorrhagic and hemolytic disorders, and assessment of the effects of chronic diseases [37]. The relationship between the hemoglobin concentration and refractive index is as follows [38]:

(6) $$n=1.38+\frac{H-H_{normal}}{5766.5}$$

where $H_{normal}$ is 140 g/L, and *H* represents the concentration of the sample measured. We set the concentration of the sample from 50 g/L to 250 g/L in steps of 50 g/L. The detection process requires injecting the test sample into the MIM waveguide tank, which changes the refractive index of the medium, leading to a shift in the Fano resonance position. The sensor’s sensitivity is related to the resonant wavelength as follows:

(7) $$S_{H}=\frac{∆\lambda }{∆H}$$

Here, ∆*λ* represents the shift in the transmission spectrum, and ∆*H* denotes the change in hemoglobin concentration of the sample measured.

**Figure 10:**
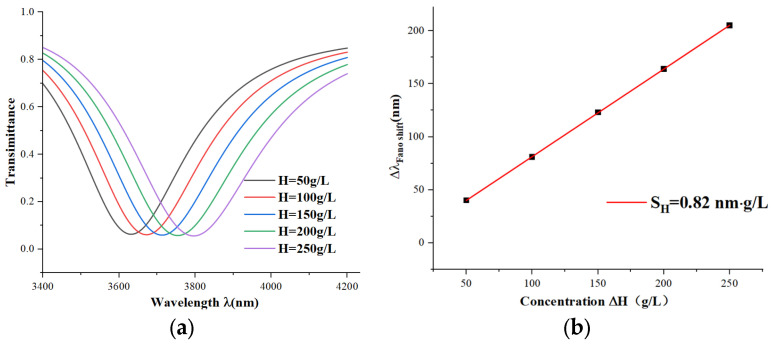
(**a**) Transmission spectra of different concentrations of H; (**b**) sensitivity fit curves for different hemoglobin concentrations.

The transmission spectrum and the sensitivity fitting curve were finally obtained, as shown in Figure 10a. The wavelength of the Fano resonance increases almost equally with the increase of the measured sample concentration, with a spacing of 41 nm. In Figure 10b, we can see the fitting curve of the sensitivity, and the linear correlation coefficient is greater than 0.999. Thus, we estimated the sensitivity of the sensor as 0.82 nm∙g/L based on our simulations. Commercial spectral analyzers have a minimum resolution of 0.001 nm, indicating that high-precision nanoscale hemoglobin concentration monitoring has great potential for development. Compared with commonly used techniques for detecting the hemoglobin concentration in blood—such as colorimetry (low sensitivity, many interfering factors, and a need for pretreatment), spectrometry (a need for pretreatment), turbidimetry (lower sensitivity and a need for a standard curve), and blood gas analyzers (a need for professional operation and high sample requirements)—this sensor has significant advantages in terms of sensitivity, precision, and real-time detection. However, the sensor is sensitive to environmental changes (e.g., temperature and hydrodynamic conditions), which may affect the stability of the results and may not guarantee high performance and accuracy for large-scale screening applications.

## 5. Conclusions

In this paper, a nanoscale refractive index sensor structure consisting of a MIM waveguide and a CMSICPRC-structured cavity is designed. The propagation characteristics of the overall structure are analyzed by experimental comparison using the finite element method (FEM), determining the optimal CMSICPRC structure. This structure maximizes the advantages of the coupling of the optical Fano resonance and the plasma waveguide. The experimental results show that the overall performance is optimal when a is 20 nm and g is 10 nm. The sensor sensitivity increases with the increase of L, and h has the weakest and almost no effect on the sensor’s performance. That is, when a = 20 nm, g = 10 nm, L = 220 nm, R = 190 nm, and h = 50 nm, according to our simulation results, the optimal sensitivity of the sensor is 3240 nm/RIU, the FOM is 50.3, and this sensor can be used in the biomedical field to detect the concentration of erythrocytes in the blood, and according to our calculations, can be 0.82 nm∙g/L. This data facilitates the diagnosis of medical conditions in clinical medicine. In addition, different blood types have varying concentrations of hemoglobin, correlating with red blood cell levels. This provides a basis for blood type recognition and supports nanosensor research in biomedical and clinical areas.

## Data Availability

The data provided in this study are available upon request from the corresponding author.
